# Supplementary material for: Wind-driven upwelling of iron sustains dense blooms and food webs in the eastern Weddell Gyre
Source: Nat Commun. 2023 Mar 9;14:1303. doi: 10.1038/s41467-023-36992-1 (PMC9998654; doi:10.1038/s41467-023-36992-1)
Supplement: Supplementary file 1 — Supplementary Information [file 41467_2023_36992_MOESM1_ESM.pdf]

Supplementary Information

Supplementary Figure 1

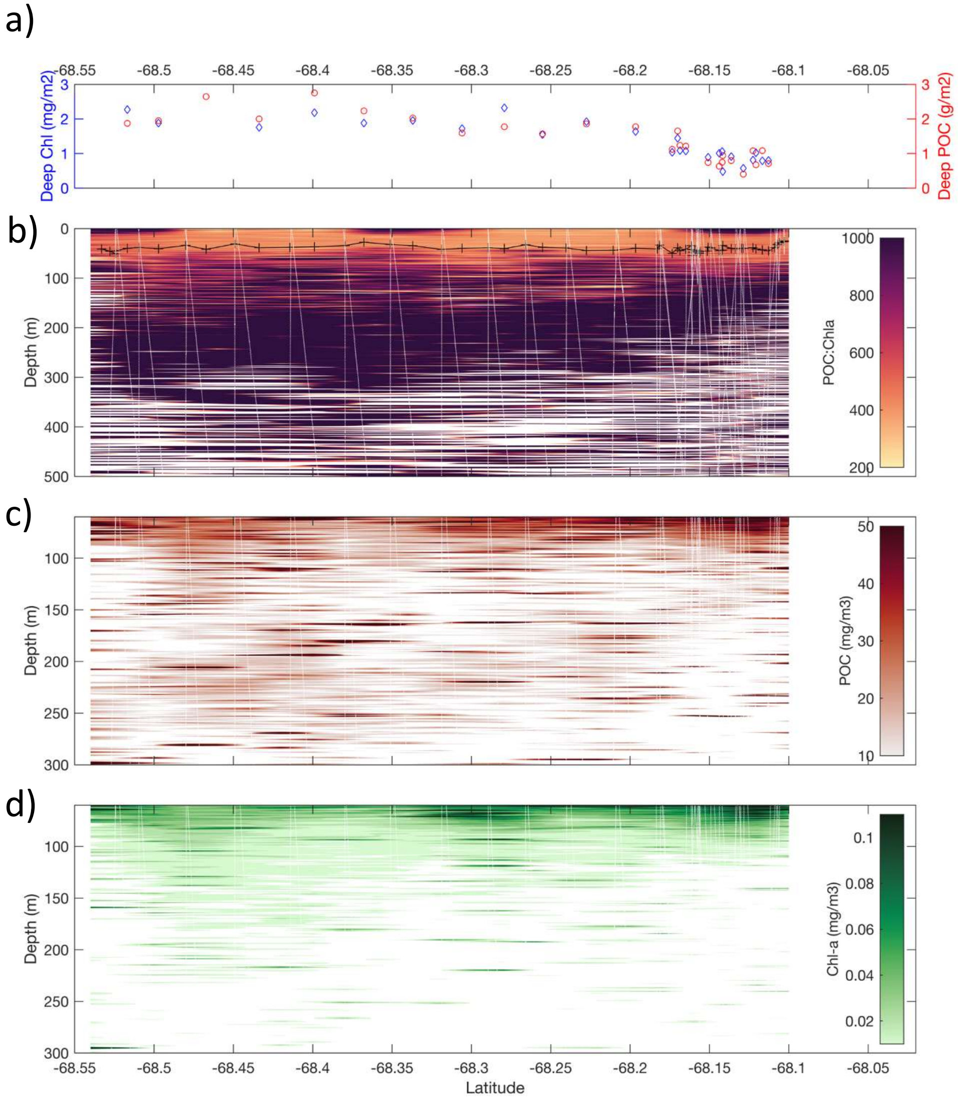

Supplementary Figure 1: Deep export from the phytoplankton bloom. a) Integrated Particulate Organic Carbon (POC, g m^{-2}) and Chl *a* (mg m^{-2}) from each glider profile between 100 and 500 m depth (the location of the glider profiles is given in Fig. 1a); b) contoured POC:Chl *a* ratio (mg C m^{-3}/mg Chl *a* m^{-3}) between the surface and 500 m depth; c) POC concentration (mg m^{-3}) between 50 and 300 m depth; d) Chl *a* concentration (mg m^{-3}) between 50 and 300 m depth.

Supplementary Figure 2

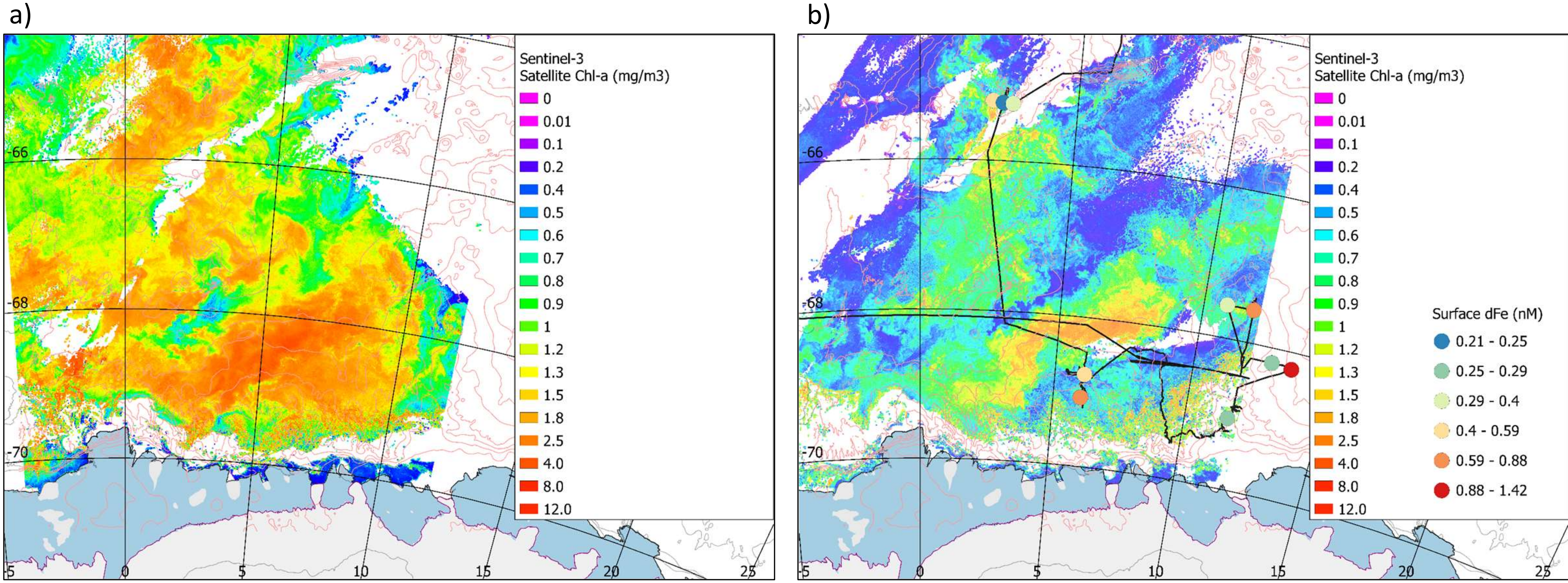

Supplementary Figure 2: Peak of the open ocean bloom and surface iron concentration. a) Sentinel satellite image of the open ocean phytoplankton bloom on February 17th, 2019. b) Sea surface dissolved iron (dFe) concentration (nM). In b), the Sentinel satellite image of the open ocean phytoplankton bloom on March 8th, 2019 is used.

Supplementary Figure 3

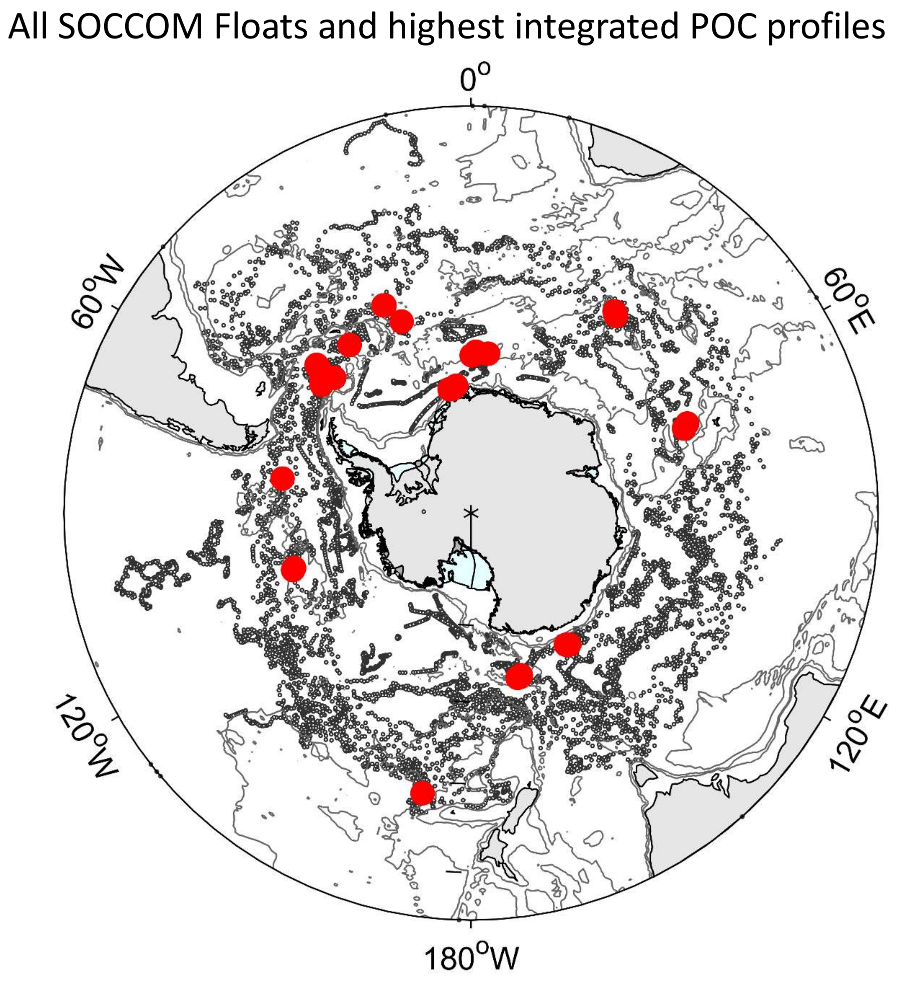

Supplementary Figure 3: POC stocks measured in the upper 100 m of the Southern Ocean by SOCCOM BGC-Argo floats. All SOCCOM BGC-Argo floats profiles that measured upper ocean POC concentration in the Southern Ocean[22] from September 2014 to December 2020, i.e., more than 9500 profiles, (black dots). The highest integrated POC stocks, where the upper 100 m integrated POC was above 15 g C m^{-2} are plotted as unique size red circles.

Supplementary Figure 4

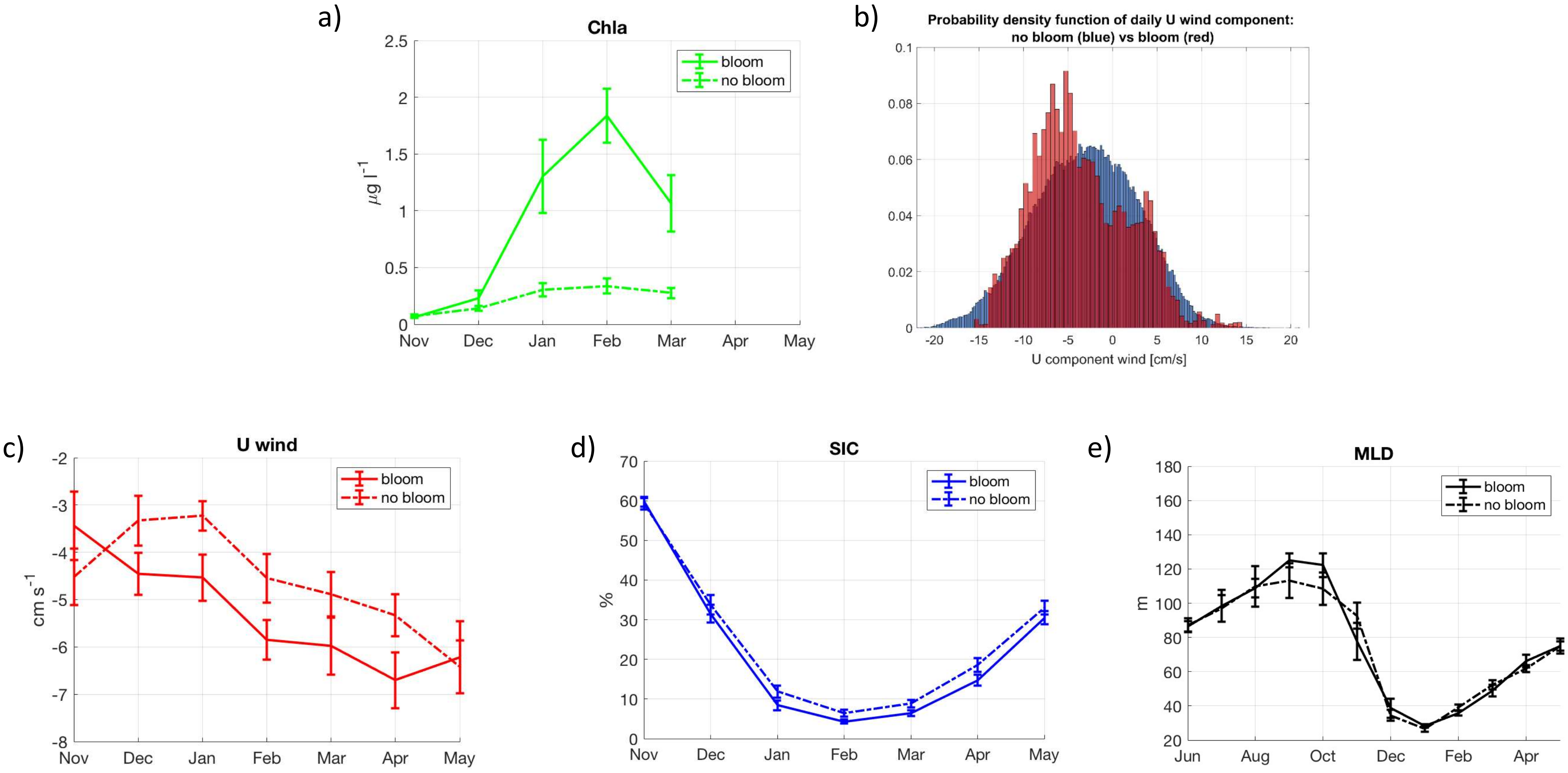

Supplementary Figure 4: Monthly mean seasonal cycles over the bloom area. a) Monthly mean seasonal cycle of the satellite-derived Chl *a* (mg m^{-3}, N = 240) over the bloom area in the presence and absence of a bloom. b) Probability density function of daily zonal wind component over the bloom area in the presence and absence of a bloom. Monthly mean seasonal cycle of c) the zonal wind (cm s^{-1} , N = 240), d) the sea ice concentration (% , N = 240), and e) the mixed layer depth (m , N = 240) over the bloom area in the presence and absence of a bloom. Data are presented as mean values ± standard error.

Supplementary Figure 5

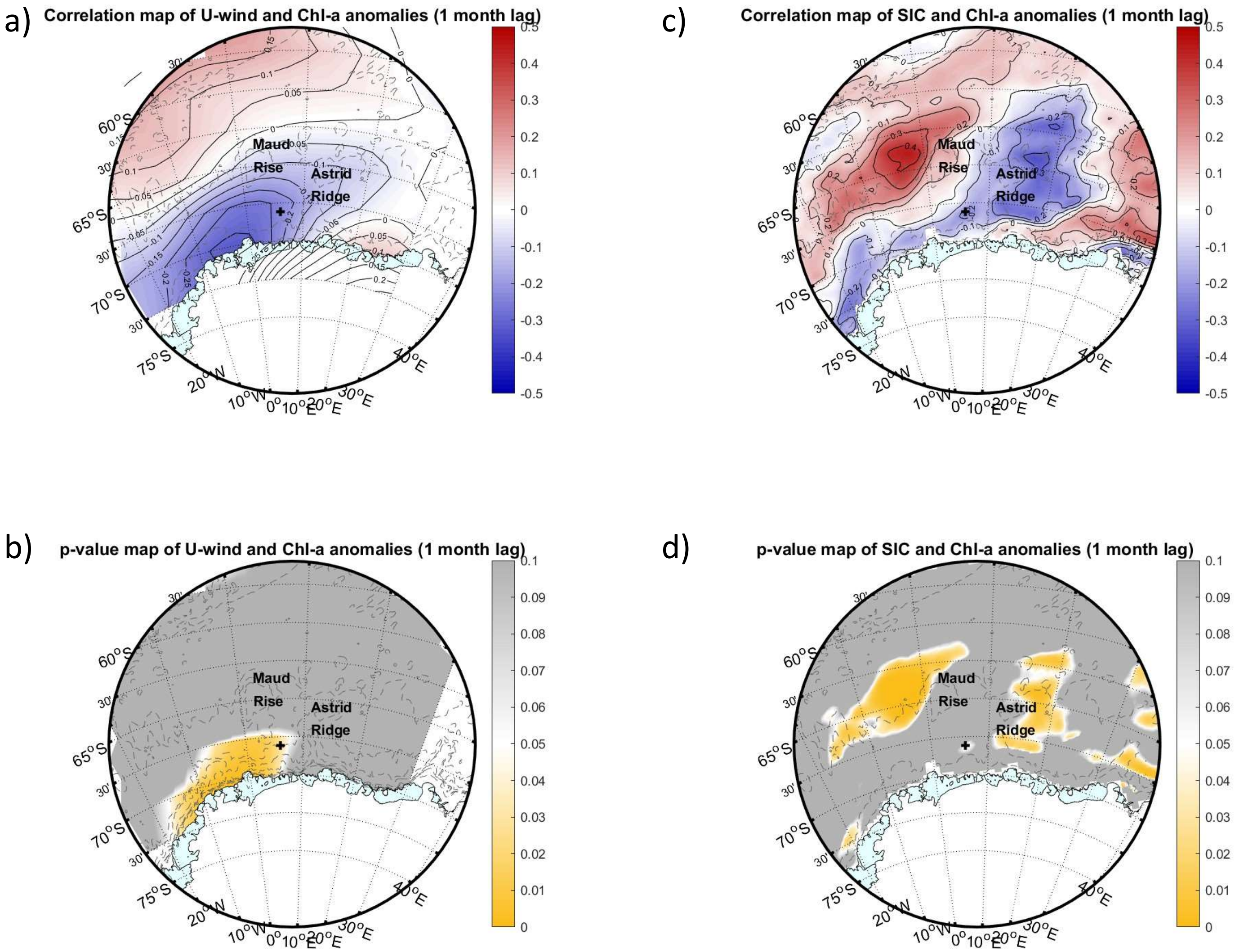

Supplementary Figure 5: Correlations between the Chl *a*, zonal wind and sea ice concentrations anomalies in the area of the bloom. a) Correlation map and b) p-value between the satellite-derived Chl *a* de-seasoned anomaly (lagged by 1-month) and the zonal wind de-seasoned anomaly in the area of the open ocean bloom. c) Correlation map and d) p-value between the satellite-derived Chl *a* de-seasoned anomaly (lagged by 1-month) and the sea ice concentration de-seasoned anomaly in the area of the open ocean bloom. Maps of associated p-values mark the areas where the correlation is significant.

Supplementary Figure 6

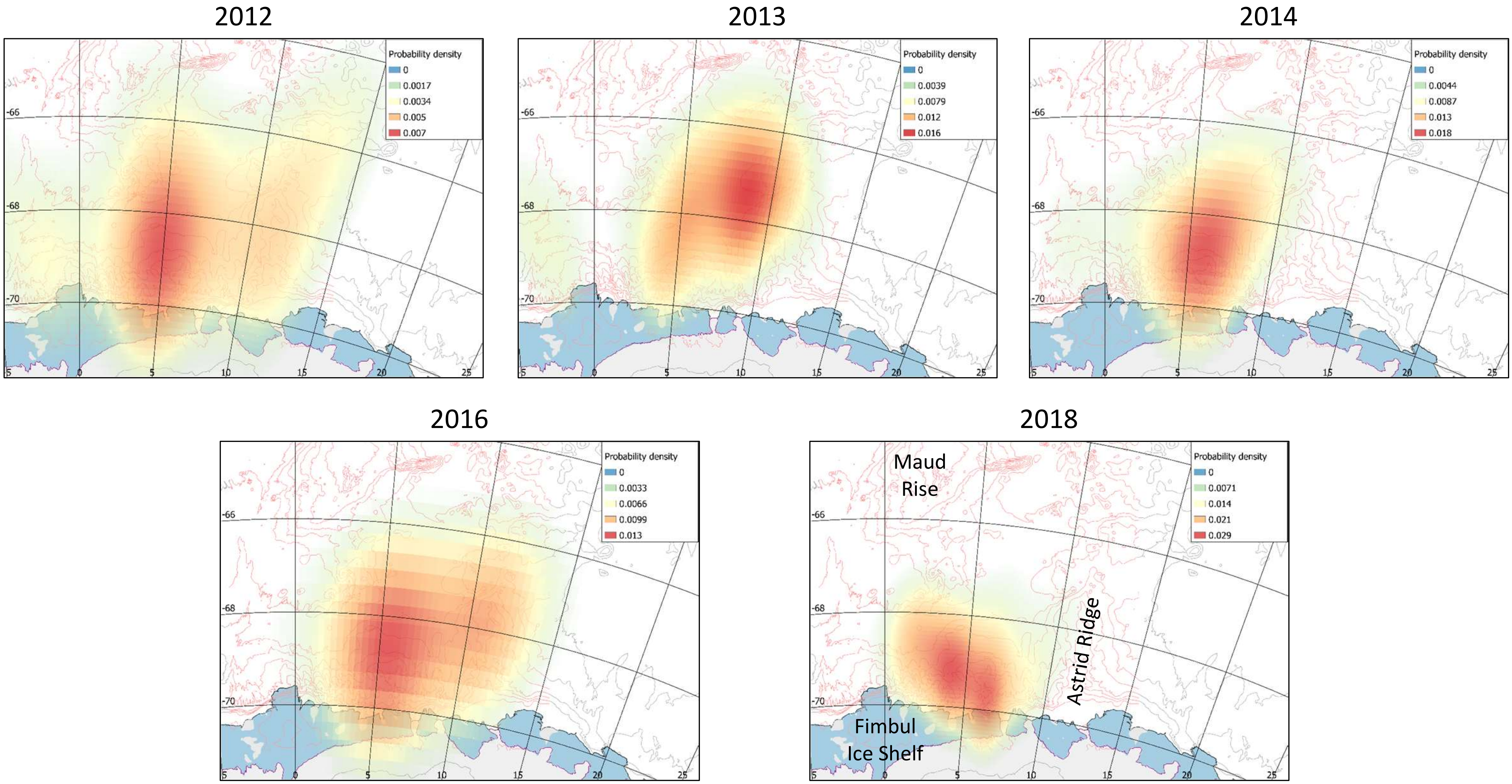

Supplementary Figure 6: Svarthamaren Antarctic petrels' distribution at sea during summer and fall. Heat map of the utilization distribution, (i.e., the probability density that an animal is found at a point according to its geographical coordinates) of Svarthamaren Antarctic petrels during January/February 2012, 2013, 2014, 2016 and 2018.

Supplementary Table 1: Demographic properties of *E. superba* in two trawl catches within the bloom area (Station 4410: 6.72ºE, -68.65ºS, 35-67 m depth and Station 4412: 3.59ºE, -68.21ºS, 36-98 m depth). Proportions (%) of sexual maturity stages, sex ratio average lengths.

**Station**	**4410**	**4412**
**Juveniles %**	3.9	6.5
**Male subadult %**	39.9	13.6
**Male adult %**	45.8	19.5
**Female subadult %**	2.0	21.4
**Female adult %**	8.5	39.0
**Sex ratio (M:F)**	8:1	1:2
**Average length (mm +/-SD)**	52.1 +/-3.3	49.9 +/- 5.1
