# Supplementary material for: Wind-driven upwelling of iron sustains dense blooms and food webs in the eastern Weddell Gyre
Source: Nat Commun. 2023 Mar 9;14:1303. doi: 10.1038/s41467-023-36992-1 (PMC9998654; doi:10.1038/s41467-023-36992-1)
Supplement: Supplementary file 3 — Reporting Summary [file 41467_2023_36992_MOESM3_ESM.pdf]

nature portfolio

Corresponding author(s): Sebastien Moreau

Last updated by author(s): 2022_12_30

Reporting Summary

Nature Portfolio wishes to improve the reproducibility of the work that we publish. This form provides structure for consistency and transparency in reporting. For further information on Nature Portfolio policies, see our Editorial Policies and the Editorial Policy Checklist.

Statistics

For all statistical analyses, confirm that the following items are present in the figure legend, table legend, main text, or Methods section.

n/a	Confirmed	
[ ]	[x]	The exact sample size (*n*) for each experimental group/condition, given as a discrete number and unit of measurement
[ ]	[x]	A statement on whether measurements were taken from distinct samples or whether the same sample was measured repeatedly
[ ]	[x]	The statistical test(s) used AND whether they are one- or two-sided<br>*Only common tests should be described solely by name; describe more complex techniques in the Methods section.*
[ ]	[x]	A description of all covariates tested
[ ]	[x]	A description of any assumptions or corrections, such as tests of normality and adjustment for multiple comparisons
[ ]	[x]	A full description of the statistical parameters including central tendency (e.g. means) or other basic estimates (e.g. regression coefficient) AND variation (e.g. standard deviation) or associated estimates of uncertainty (e.g. confidence intervals)
[ ]	[x]	For null hypothesis testing, the test statistic (e.g. *F*, *t*, *r*) with confidence intervals, effect sizes, degrees of freedom and *P* value noted<br>*Give P values as exact values whenever suitable.*
[x]	[ ]	For Bayesian analysis, information on the choice of priors and Markov chain Monte Carlo settings
[x]	[ ]	For hierarchical and complex designs, identification of the appropriate level for tests and full reporting of outcomes
[ ]	[x]	Estimates of effect sizes (e.g. Cohen's *d*, Pearson's *r*), indicating how they were calculated

Our web collection on statistics for biologists contains articles on many of the points above.

Software and code

Policy information about availability of computer code

Data collection	The utilization distribution maps of Antarctic petrels (or heat maps) were created with the kernelUD function of package adehabitatHR in R software version 4.0.2, using the href (reference bandwidth) smoothing parameter (https://www.rdocumentation.org/packages/adehabitatHR/versions/0.4.19/topics/kernelUD).<br><br>The abundance of krill was studied with a Simrad EK80 research echosounder with six frequencies. For this study, we focused on the 38 kHz frequency. It was scrutinized with the Large Scale Survey System (LSSS) software version 2.5.0.<br><br>The samples were measured using a Waters HPLC system (Waters Corporation, Milford MA, USA) equipped with an autosampler (model 717 plus autosampler), an HPLC pump (model 600 HPLC LCD pump), a photodiode array detec- tor (model PDA 2996), a fluorescence detector (model 2475 fluorescence detector) and the Empower 3 Chromatography Data Software (CDS).
Data analysis	Data analyses post field work were conducted with Matlab R2019b. All maps were built on QGIS (v. 3.20.0) and using the m_map package as downloaded from https://www.eoas.ubc.ca/~rich/map.html in November 2021.

For manuscripts utilizing custom algorithms or software that are central to the research but not yet described in published literature, software must be made available to editors and reviewers. We strongly encourage code deposition in a community repository (e.g. GitHub). See the Nature Portfolio guidelines for submitting code & software for further information.

Data

Policy information about availability of data

All manuscripts must include a data availability statement. This statement should provide the following information, where applicable:

- Accession codes, unique identifiers, or web links for publicly available datasets
- A description of any restrictions on data availability
- For clinical datasets or third party data, please ensure that the statement adheres to our policy

The Seaglider, the biogeochemical and the taxonomical data from the scientific campaign can be found at the Norwegian Polar Data Centre (Norwegian Polar Institute): https://data.npolar.no/dataset/ab96f43d-b813-4457-a466-6982c2c60a6b, https://data.npolar.no/dataset/28fbddd2-0fb2-41c9-9f42-60146e28617f and https://data.npolar.no/dataset/283e500c-732b-4f9b-a48a-3bc4990e3f55. In addition, the marine birds and mammals distribution data can be found at https://data.npolar.no/dataset/5168ad7f-4733-45fd-87bd-4c4c4c217876.

Atmospheric reanalyses can be obtained from https://www.ecmwf.int/en/forecasts/datasets/reanalysis-datasets/era-interim. Satellite derived ocean color is available at https://oceandata.sci.gsfc.nasa.gov/ and https://www.eumetsat.int/ocean-colour-services. Satellite derived sea ice concentration is available at http://nsidc.org. The SOCCOM data are available at https://soccom.princeton.edu. The GLODAP Version 2 data product can be obtained from https://www.glodap.info/. Bird tracking data (2012-2016) can be found at https://www.datarepository.movebank.org/handle/10255/move.566.

Human research participants

Policy information about studies involving human research participants and Sex and Gender in Research.

Reporting on sex and gender	This information has not been collected
Population characteristics	This information has not been collected
Recruitment	This information has not been collected
Ethics oversight	This research involved no human research participants

Note that full information on the approval of the study protocol must also be provided in the manuscript.

Field-specific reporting

Please select the one below that is the best fit for your research. If you are not sure, read the appropriate sections before making your selection.

☐ Life sciences ☐ Behavioural & social sciences ☒ Ecological, evolutionary & environmental sciences

For a reference copy of the document with all sections, see nature.com/documents/nr-reporting-summary-flat.pdf

Ecological, evolutionary & environmental sciences study design

All studies must disclose on these points even when the disclosure is negative.

Study description	In this study, we report on a considerably dense late summer phytoplankton bloom spanning 9,000 km2 in the open ocean of the Kong Håkon VII Hav, eastern Weddell Gyre. Over its two and a half months duration, this phytoplankton bloom accumulated high levels of organic matter, up to 20 g C m-2. We show that the bloom presence is linked to anomalies in easterly winds that push sea ice southwards and favor the upwelling of iron-enriched Warm Deep Water fueling phytoplankton production. Deep hydrothermal iron is likely delivered to the bloom region by the northern pathway of Warm Deep Water into the gyre, as is supported by locally enhanced concentrations of primordial helium. This recurring open ocean bloom sustains high standing stocks of Antarctic krill, supporting feeding hot spots for top trophic level consumers such as baleen whales and marine birds.
Research sample	This study relies on in situ data collected during an oceanographic cruise. The variables used in the study are: particulate organic matter, phytoplankton pigments, phytoplankton taxonomy, dissolved oxygen isotopes, dissolved iron, helium isotopes, and krill abundance. The study also uses at-sea observations of marine mammals and seabirds. Furthermore, the study also relies on using long-term satellite data of ocean color, sea ice cover, and winds as well as oceanographic data retrieved from a Seaglider and SOCCOM floats. These data are meant to represent the typical biogeochemistry and food-web of the Southern Ocean, from the base of the food-web (i.e., phytoplankton), to its main grazers (i.e., krill) and the top of the food chain (marine birds and baleen whales). The phytoplankton (Chaetoceros dichaeta), krill (juveniles and adult males and females Euphausia superba), marine birds (breeding adults Thalassoica antarctica and Pagodroma nivea) and whales (feeding female and male calves and adults Megaptera novaeangliae) correspond to the major species that constitute the typical Southern Ocean food web. No manipulations of the organisms was done and only krill individuals were collected.
Sampling strategy	The sampling strategy was decided opportunistically at sea upon the discovery of this large phytoplankton bloom with satellite derived ocean color images. Therefore, no sample size was pre-determined. However, the Seaglider data provided an ample dataset of the blooming waters (with more than 30 profiles from the sea surface down to more than 1,000 meters depth). The satellite data

	spans over 1997-2020, and provides an ample dataset to analyze the reoccurence of this bloom and its environmental drivers over the last 2 decades. The at-sea distribution of higher trophic levels spans over 40 days at sea and comprises data gathered throughout the day, weather permitting, thus providing an ample dataset to study the distribution of higher trophic levels throughout this region of the Southern Ocean.
Data collection	The data collection was performed following the international scientific standards for oceanographic and marine biology studies. The oceanographic data were recorded with a Seaglider and a CTD-rosette and analyzed by Sebastien Moreau, Hanna Kauko, Asmita Singh, Thomas Ryan-Keogh, Tore Hattermann, Laura de Steur, Nadine Steiger, Melissa Chierici, Agneta Fransson, Pedro Monteiro, Sandy Thomalla, Murat Ardelan, Nicolas Sanchez, Alakendra Roychoudhury, Ilka Peeken, Philipp Assmy, Anais Lebrun, Magdalena Rozanska and Jozef Wiktor. The krill data were recorded with a krill trawl and analyzed by Tone Falkenhaug, Jan Henrik Simonsen and Elvar Hallfredsson. The at-sea distribution of higher trophic levels were recorded visually and analyzed by Eirik Grønningsæter, Andre van Tonder, Nico Lübcker, Andrew Lowther, Heidi Ahonen, Sebastien Descamps and Harald Steen.
Timing and spatial scale	The study was done in March 2019 during the Southern Ocean Ecosystem 2019 cruise on board the RV Kronprins Haakon in the Kong Håkon VII Hav, eastern Weddell Gyre,
Data exclusions	No data were excluded from this study
Reproducibility	This research is not based on an experiment but on raw data obtained in the field. Therefore, while the data collection is a one time event that cannot be reproduced, we tested successfully that the data analysis can be reproduced from the publicly available data.
Randomization	This study is based on oceanographic and marine biology data collected in situ. Since the data presented in this study are not part of an experiment, data randomization is not necessary to the presentation of the data.
Blinding	This study is based on oceanographic and marine biology data collected in situ. Since the data presented in this study are not part of an experiment, blinding was not necessary.

Did the study involve field work? ☒ Yes ☐ No

Field work, collection and transport

Field conditions	The study was done in March 2019 in the Kong Håkon VII Hav, eastern Weddell Gyre, which is fall in the Southern Ocean, a time of the year when air temperature decrease to below zero degrees Celcius. Decreasing photoperiod (i.e. down to 14h per day), snow precipitation and sea ice formation occurred during the cruise.
Location	The study was done in the Kong Håkon VII Hav, eastern Weddell Gyre, so between 0 and 10 degrees East and -66 to -70 degrees. South. The study describes a considerably dense late summer phytoplankton bloom spanning 9,000 km2 in the open ocean of the Kong Håkon VII Hav, over depth of 3,000 m deep.
Access & import/export	Data were collected in a responsible manner following the international recommendations of the Antarctic Treaty System. The data were collected with a non-invasive Seaglider as well as with a CTD-rosette and a krill trawl on board the RV Kronprins Haakon. At any time, the scientific teams elaborated maximum efforts to avoid destructive sampling of the environment. For example a minimum volume of seawater was collected in each Niskin bottle (12L) for all biological and biogeochemical analyses, and a minimum number of krill individuals (i.e., ~150) were collected for krill analyses. Samples were subsequently imported to Norway on board the RV Kronprins Haakon for onshore laboratory analyses. A permit was obtained by the Norwegian Polar Institute in late 2018 for the Southern Ocean Ecosystem 2019 cruise on board the RV Kronprins Haakon in the Kong Håkon VII Hav.
Disturbance	Minimal disturbance was caused by the presence of the ship in the area.

Reporting for specific materials, systems and methods

We require information from authors about some types of materials, experimental systems and methods used in many studies. Here, indicate whether each material, system or method listed is relevant to your study. If you are not sure if a list item applies to your research, read the appropriate section before selecting a response.

Materials & experimental systems

n/a	Involved in the study	
☒	☐	Antibodies
☒	☐	Eukaryotic cell lines
☒	☐	Palaeontology and archaeology
☐	☐	Animals and other organisms
☒	☐	Clinical data
☒	☐	Dual use research of concern

Methods

n/a	Involved in the study	
☒	☐	ChIP-seq
☒	☐	Flow cytometry
☒	☐	MRI-based neuroimaging

Animals and other research organisms

Policy information about studies involving animals; ARRIVE guidelines recommended for reporting animal research, and Sex and Gender in Research

Laboratory animals	One animal involved in the study was Antarctic krill, Euphausia superba , samples of which were obtained with trawls in the area of the open ocean bloom. In addition, this study reports about previous data on the distribution at sea of Antarctic petrels tagged at Svarthamaren, Antarctica.
Wild animals	Krill swarms were sampled with a Macroplankton trawl at two locations within the bloom region. The trawl is a fine-meshed plankton trawl with a 36 m2 mouth-opening and 3x3 mm diamond shaped mesh (7 mm stretched) from mouth to rear. From each trawl, a subsample of approximately 150 individuals of Euphausia superba was taken, and the length of the individual krill was measured (+/-1 mm) from the anterior margin of the eye to tip of telson excluding the setae. The 150 individuals were frozen for further analyses ashore. The remaining krill individuals were released.<br><br>Regarding the tagging of Antarctic petrels at Scarthamaren, Antarctica, breeding adults were captured on theirs nest during late incubation and mid chick rearing (from early January to mid-February) and instrumented with Global Positioning System (GPS) loggers (CatTrack 1, Catnip Technologies Ltd., Anderson, USA) just before leaving on a foraging trip. The GPS units weighed approx. 20 g (ca. 3% of bird body mass) and were taped to tail feathers. Birds were recaptured upon return to their nest to retrieve the GPS units and download the data. GPSs recorded the locations of the birds along their foraging trip at 5 to 30 min intervals. No animals were harmed in the process.
Reporting on sex	Sex and maturity stages of E. superba were determined using the classification methods according to Makarov and Denis (1981). For Antarctic petrels, breeding adults were captured on theirs nest during late incubation and mid chick rearing (from early January to mid-February) and instrumented with Global Positioning System (GPS) loggers just before leaving on a foraging trip.
Field-collected samples	No experiments were performed on the sampled krill or the Antarctic petrels.
Ethics oversight	The organization of the field work was done by the Norwegian authorities (Fisheries Ministry and Ministry of Foreign Affaires) under the science guidance of the Norwegian Polar Institute and the Institute of Marine Research and following international law and recommendations set by the Antarctic Treaty System.

Note that full information on the approval of the study protocol must also be provided in the manuscript.
